# Rheumatoid Arthritis Treatment. A Back to the Drawing Board Project or High Expectations for Low Unmet Needs?

**DOI:** 10.3390/jcm8081237

**Published:** 2019-08-16

**Authors:** Alexandros A. Drosos, Eleftherios Pelechas, Paraskevi V. Voulgari

**Affiliations:** Rheumatology Clinic, Department of Internal Medicine, Medical School, University of Ioannina, 45110 Ioannina, Greece

**Keywords:** rheumatoid arthritis treatment, unmet needs, treat to target, ACR/EULAR recommendations

## Abstract

Despite the significant progress in Rheumatoid Arthritis (RA) therapeutics, there are several reports in the literature claiming that the size of unmet needs in RA is large. In the era before biologics, there was indeed a significant number of patients who did not achieve low disease activity (LDA) or disease remission due to limited therapeutic choices in the doctors’ armamentarium. Treatment wise, great progress has been achieved over the last decades with the discovery and introduction in therapeutics of new molecules, such as the biological (b) disease-modifying anti-rheumatic drugs (DMARDs), and the targeted synthetic (ts) DMARDs. Today, with such a plethora of conventional synthetic (cs) DMARDs, tsDMARDs, and bDMARDs, why are we unable to successfully treat RA patients? What is wrong? However, a new drug for RA does not mean it is necessary to switch to a new treatment. It is very easy to change and switch therapies when the patient complains about pain and stiffness. In this setting, it is obligatory to rule out other comorbidities and disorders that may be the cause of the pain first. Thus, clinicians must have a deep knowledge of the drug therapy and be able to adjust the treatment when needed. A minute clinical examination must be carried out on every visit with close monitoring of the patient. A treat-to-target (T2T) approach and the application of the American College of Rheumatology/European League Against Rheumatism (ACR/EULAR) recommendations and strategies should minimize the unmet needs.

## 1. Introduction

Rheumatoid arthritis (RA) is a chronic inflammatory disease affecting the peripheral skeleton with a predilection for the small joints of the hands and feet in a symmetrical manner [1]. The occurrence, in the Caucasian populations, has been estimated to be between 0.5 to 1% [2,3]. If the disease remains untreated, it can cause severe joint damage, bone destruction, poor quality of life, and high morbidity and mortality [4]. Thus, early diagnosis and early intervention are imperative. To this end, the 1987 American College of Rheumatology (ACR) and the ACR/European League Against Rheumatism (EULAR) 2010 classification criteria helped physicians to improve their diagnostic accuracy [5,6]. Treatment wise, significant progress has been achieved over the last two decades. The introduction of biologic disease-modifying antirheumatic drugs (bDMARDs) targeting cytokines, such as tumor necrosis factor-α (TNF-α) [7,8], the interleukin (IL)-6 receptor [9], co-stimulatory molecules of T-cells [10] or, other molecules of B-cells [11], has revolutionized RA management. Recently, targeted synthetic (ts) DMARDs inhibiting the Janus kinases (JAK) have also been approved [12,13]. In addition, new treatment strategies and recommendations from the ACR, EULAR and other institutions are promising interventions which imply a new era for RA management. Sustained clinical remission, low disease activity (LDA), halting the progression of joint damage, and improving patients’ quality of life are some of the results of these interventions [14,15,16]. Finally, new tools for monitoring disease activity have been created, such as the ACR 20/50/70 clinical response criteria as well as the disease activity score for 28 joints (DAS-28) and others [17,18]. However, despite all this multilevel approach and progress in the field for RA diagnosis, monitoring and treatment, there are several reports in the literature showing that a significant number of RA patients does not achieve remission or LDA. This information comes from European and American registries as well as expert opinions, suggesting that there are still significant unmet needs for RA treatment [19,20,21,22,23,24,25].

## 2. Matters Arising

After that, many questions arise. Is there indeed such gap and huge unmet needs in RA management? Are the data from registries and expert opinions strong enough to suggest such high unmet needs for RA management? Are we still in need of more long-term and better structured observational studies to clarify the subject? On the other hand, do all physicians follow the ACR/EULAR recommendations for RA management and the treat-to-target (T2T) approach? Does training and education in rheumatology differ among countries?

All the above questions arose because back in the 1980s, we only had in our therapeutic armamentarium some conventional synthetic (cs) DMARDs, such as methotrexate (MTX), sulfasalazine (SSZ), hydroxychloroquine (HCQ), d-penicillamine, gold salts, and used them as monotherapy or in combination with steroids [26,27,28]. Cyclosporine-A and leflunomide (LFN) were approved in the 1990s [29]. Back then, treating RA was a difficult task, and indeed, many patients had an inadequate response leading to real unmet needs in RA treatment. However, over the last years we use the majority of csDMARDs used in the past (MTX, LFN, SSZ, HCQ), five biologics targeting the TNF-α (adalimumab, certolizumab, etanercept, golimumab, and infliximab), one biologic targeting the IL-6 receptor and another one targeting the IL-1, one targeting the co-stimulatory molecule cytotoxic T-lymphocyte-associated antigen 4 (CTLA4), and one targeting the CD20 molecule of B-cells. In addition, we have two tsDMARDs targeting the JAK kinases. Finally, four TNF-a biosimilars and one targeting B-cells are already in the market [30,31,32]. With such a plethora of cs, ts, bDMARDs, and biosimilars, why we are unable to successfully treat RA patients? Which of the above questions are true, and which are false? What is wrong?

## 3. Treatment Strategies

Treatment decisions and strategies are very crucial to obtain a rapid disease control and to achieve, as early as possible, remission or LDA. To this end, the first approach should include early and correct diagnosis, as well as early intervention. The second step should include all the above-mentioned cs, b, and tsDMARDs in an appropriately designed manner for each patient. In this direction, the T2T strategy is the ideal approach. T2T therapy comprises fine principles: (a) the definition of a treatment target, (b) close monitoring and follow-up at predefined times to assess disease activity using composite measures, (c) regular treatment adjustment if the target is not achieved within a scheduled time, (d) consideration of patients’ individualized aspects, and (e) the shared decision making with the patient [14,33,34]. MTX is considered the “anchor”-drug among the csDMARDs. It is recommended as monotherapy, as a step-up combination therapy with other csDMARDs, or as an initial csDMARDs combination therapy. The most popular csDMARDs combination of MTX is MTX plus HCQ and the triple combination of MTX, plus HCQ and SSZ. Rapid disease control can be achieved by using steroids (prednisone) as a “bridging” therapy but dose tapering <7.5 mg/day is essential to avoid and to minimize the side effects. Therapeutic decisions should be guided by safety, tolerability of the drugs, but also cost-effectiveness [34].

Despite the many advantages of the csDMARDs, especially of MTX, they may still be used sub-optimally. Indeed, studies indicate that half of the RA patients discontinued MTX within the first 2 years of treatment and that MTX is frequently stopped rather than combined with other cs, b, or tsDMARDs [24]. Triple therapy combining MTX, HCQ, and SSZ may be a valuable alternative to the addition of b or tsDMARDs to MTX, in cases of inadequate response to MTX and in the absence of poor prognostic factors [35,36,37].

## 4. Early RA Treatment

Recently, Kaltsonoudis et al. [38] reported that treating early RA patients with all the available csDMARDs or/and bDMARDs, following the T2T approach and the ACR/EULAR recommendations and strategies for RA, were able to treat and achieve LDA in the majority of the patients. In this study, 66% (first group) of patients were treated with csDMARDs as monotherapy or in combination therapy, plus small doses of steroids, and 34% (second group) were receiving bDMARDs with or without csDMARDs. After 12 years of follow-up, 3.2% of patients from the first group and 17.7% of the second group had an inadequate response to all treatment options and never achieved LDA. Thus, the unmet needs for RA treatment in this cohort was 20.9%. This is a reasonable size of unmet needs for RA management, taking into account the long-term follow-up [38]. In a prospective cohort study in early RA patients, investigators from the COBRA (Combinatietherapie Bij Reumatoide Artritis) study compared csDMARD combination plus a high dose of steroids versus monotherapy. It was shown that COBRA combination treatment was superior to monotherapy in disease control with less adverse events [39]. After 5 years of follow-up, the same investigators demonstrated that combination therapy maintained a better disease control with less radiological progression [40]. In addition, after 11 years of follow-up, the same patients using combination therapy had lower mortality rates compared to those on monotherapy [41]. Finally, after 23 years of follow-up using the T2T approach, the COBRA trial demonstrated that early RA patients had normalized mortality rates as compared to the general population [42]. The above studies confirm that early and intensive treatment approach has long-term beneficial results, not only in lowering disease activity and reducing structural damage progression but also in reducing morbidity and mortality.

What have we learned from the above studies? All were long-term observational studies treating early RA patients applying T2T approach. The majority of the patients in the Kaltsonoudis et al. study [38] and all patients in the COBRA studies [39,40,41,42] were treated with csDMARDs in combination, plus steroids. Finally, all patients had a close follow-up and monitoring. Several other studies are using triple csDMARDs in combination versus bDMARDs + MTX, showed no differences between the two groups. However, we do not imply that csDMARDs are superior to bDMARDs, but when the csDMARDs are used early in the course of RA and in combination, then the results are comparable to those receiving bDMARDs [43,44,45,46]. To this end, new prospective long-term observational studies from early arthritis centers following the T2T approach and the ACR/EULAR recommendations for RA management would be useful to clarify and to demonstrate the real size of unmet needs in RA treatment.

All the above studies teach us that RA treatment needs reasonable management with a deep knowledge of the drug therapy adjusting therapy according to the clinical response, a minute clinical examination of every patient on every visit and finally change therapy when indicated. It is very easy to change and switch therapies when the patient is complaining about pain and stiffness. In this setting, there is no immediate need to discontinue treatment or change therapy. It is obligatory to rule out other comorbidities and disorders which may be the cause of the pain, such as tendinitis, carpal tunnel syndrome, fibromyalgia, or depression, first.

## 5. Conclusions

Investigators and the pharmaceutical companies will continue to discover new molecules, and new drugs will be approved for RA management [47]. However, rheumatologists must use bDMARDs and the new drugs which are in development with caution, and to pay much more attention when the patients’ complains. Newer drugs for RA does not mean it is necessary to switch to a new treatment. Today, the therapeutic armamentarium for RA has a plethora of old and new drugs, and rheumatologists need to have skills and good knowledge of RA management. Thus, the therapeutic intervention must be: early and correct diagnosis, T2T approach following the ACR/EULAR recommendations and strategies, as well as, close follow-up and monitoring. With this approach, we expect to successfully treat the majority of our patients and minimize the unmet needs for RA treatment.

## References

[1] Pelechas E., Kaltsonoudis E., Voulgari P.V., Drosos A.A., Pelechas E. (2019). Rheumatoid Arthritis. Illustrated Handbook of Rheumatic and Musculo-Skeletal Diseases.

[2] Alamanos Y., Drosos A.A. (2005). Epidemiology of adult rheumatoid arthritis. Autoimmun. Rev..

[3] Alamanos Y., Voulgari P.V., Drosos A.A. (2006). Incidence and prevalence of rheumatoid arthritis, based on the 1987 American College of Rheumatology criteria: A systematic review. Semin. Arthritis Rheum..

[4] Dadoun S., Zeboulon-Ktorza N., Combescure C., Elhai M., Rozenberg S., Gossec L., Fautrel B. (2013). Mortality in rheumatoid arthritis over the last fifty years: Systematic review and meta-analysis. Joint Bone Spine.

[5] Arnett F.C., Edworthy S.M., Bloch D.A., McShane D.J., Fries J.F., Cooper N.S., Healey L.A., Kaplan S.R., Liang M.H., Luthra H.S. (1988). The American Rheumatism Association 1987 revised criteria for the classification of rheumatoid arthritis. Arthritis Rheumatol..

[6] Aletaha D., Neogi T., Silman A.J., Funovits J., Felson D.T., Bingham C.O., Birnbaum N.S., Burmester G.R., Bykerk V.P., Cohen M.D. (2010). 2010 rheumatoid arthritis classification criteria. An American College of Rheumatology/European League against Rheumatism collaborative initiative. Ann. Rheum. Dis..

[7] Pelechas E., Voulgari P.V., Drosos A.A. (2019). Golimumab for Rheumatoid Arthritis. J. Clin. Med..

[8] Aaltonen K.J., Virkki L.M., Malmivaara A., Konttinen Y.T., Nordstrom D.C., Blom M. (2012). Systematic review and meta-analysis of the efficacy and safety of existing TNF blocking agents in treatment of rheumatoid arthritis. PLoS ONE.

[9] Scott L.J. (2017). Tocilizumab: A Review in Rheumatoid Arthritis. Drugs.

[10] Papagoras C., Drosos A.A. (2011). Abatacept: A biologic immune modulator for rheumatoid arthritis. Expert Opin. Biol. Ther..

[11] Schioppo T., Ingegnoli F. (2017). Current perspective on rituximab in rheumatic diseases. Drug Des. Dev. Ther..

[12] O’Shea J.J., Schwartz D.M., Villarino A.V., Gadina M., McInnes I.B., Laurence A. (2015). The JAK-STAT pathway: Impact on human disease and therapeutic intervention. Annu. Rev. Med..

[13] Doiugados M., van der Jeijde D., Chen Y.C., Greenwald M., Drescher E., Liu J., Beattie S., Witt S., de la Torre I., Gaich C. (2017). Baricitinib in patients with inadequate response or intolerance to conventional synthetic DMARDs: Results from the RA-BUILD study. Ann. Rheum. Dis..

[14] Smolen J.S., Breedveld F.C., Burmester G.R., Bykerk V., Dougados M., Emery P., Kvien T.K., Navarro-Compan M.V., Oliver S., Schoels M. (2016). Treating rheumatoid arthritis to target: 2014 update of the recommendations of an international task force. Ann. Rheum. Dis..

[15] Singh J.A., Saag K.G., Bridges S.L., Akl E., Bannuru R.R., Sullivan M.C., Vaysbrot E., McNaughton C., Osani M., Shmerling R.H. (2016). 2015 American College of Rheumatology Guideline for the Treatment of Rheumatoid Arthritis. Arthritis Rheumatol..

[16] Smolen J.S., Landewe R., Bijlsma J., Burmester G., Chatzidionysiou K., Dougados M., Nam J., Ramiro S., Voshaar M., van Vollenhoven R. (2017). EULAR recommendations for the management of rheumatoid arthritis with synthetic and biological disease-modifying antirheumatic drugs: 2016 update. Ann. Rheum. Dis..

[17] Anderson J., Caplan L., Yazdany K., Robbins M.L., Neogi T., Michaud K., Saag K.G., O’Dell J.R., Kazi S. (2012). Rheumatoid arthritis disease activity measures: American College of Rheumatology recommendations for use in clinical practice. Arthritis Care Res..

[18] Prevoo M.L., van ‘t Hof M.A., Kuper H.H., van Leeuwen M.A., van de Putte L.B., van Riel P.L. (1995). Modified disease activity scores that include twenty-eight-joint counts. Development and validation in a prospective longitudinal study of patients with rheumatoid arthritis. Arthritis Rheumatol..

[19] Flouri I., Markatseli T.E., Voulgari P.V., Boki K.A., Papadopoulos I., Settas L., Zisopoulos D., Skopouli F.N., Iliopoulos A., Bertsias G.K. (2014). Comparative effectiveness and survival of infliximab, adalimumab, and etanercept for rheumatoid arthritis patients in the Hellenic Registry of Biologics: Low rates of remission and 5-year drug survival. Semin. Arthritis Rheum..

[20] Pope J., Combe B. (2013). Unmet needs in the treatment of rheumatoid arthritis. Open J. Rheumatol. Autoimmune Dis..

[21] De Hair M.J.H., Jacobs J.W.G., Schoneveld J.L.M., van Laar J.N. (2017). Difficult-to-treat rheumatoid arthritis: An area of unmet clinical need. Rheumatology.

[22] Giacomelli R., Afeltra A., Alunno A., Baldini C., Bartoloni-Bocci E., Berardicurti O., Carubbi F., Cauli A., Cervera R., Ciccia F. (2017). International consensus: What else can we do to improve diagnosis and therapeutic strategies in patients affected by autoimmune rheumatic diseases (rheumatoid arthritis, spondyloarthritides, systemic sclerosis, systemic lupus erythematosus, antiphospholipid syndrome and Sjogren’s syndrome)? The unmet needs and the clinical grey zone in autoimmune disease management. Autoimmun. Rev..

[23] Taylor P.C., Moore A., Vasilescu R., Alvir J., Tarallo M. (2016). A structured literature review of the burden of illness and unmet needs in patients with rheumatoid arthritis: A current perspective. Rheumatol. Int..

[24] Olsen I.C., Lie E., Vasilescu R., Wallenstein G., Strengholt S., Kvien T.K. (2019). Assessments of the unmet need in the management of patients with rheumatoid arthritis: Analyses from the NOR-DMARD registry. Rheumatology.

[25] Winthrop K.L., Strand V., van den Heijde D.M., Mease P.J., Crow M.K., Weinblatt M., Bathon J.M., Buch M.H., Burmester G.R., Dougados M. (2016). The unmet need in rheumatology: Reports from the Targeted Therapies meeting 2016. Clin. Exp. Rheumatol..

[26] Drosos A.A., Psychols D., Andonopoulos A.P., Stefanaki-Nikou S., Tsianos E.B., Moutsopoulos H.M. (1990). Methotrexate therapy in rheumatoid arthritis. A two year prospective follow-up. Clin. Rheumatol..

[27] Drosos A.A., Karantanas A.H., Psychos D., Tsampoulas C., Moutsopoulos H.M. (1990). Can treatment with methotrexate influence the radiological progression of rheumatoid arthritis?. Clin. Rheumatol..

[28] Drosos A.A., Georgiou P., Politi E.N., Voulgari P.V. (1997). D-penicillamine in early rheumatoid arthritis. Clin. Exp. Rheumatol..

[29] Drosos A.A., Voulgari P.V., Papadopoulos I.A., Politi E.N., Georgiou P.E., Zikou A.K. (1998). Cyclosporine A in the treatment of early rheumatoid arthritis. A prospective, randomized 24-month study. Clin. Exp. Rheumatol..

[30] Lyman G.H., Zon R., Harvey R.D., Schilsky R.L. (2018). Rationale, Opportunities, and Reality of Biosimilar Medications. N. Engl. J. Med..

[31] Pelechas E., Voulgari P.V., Drosos A.A. (2018). ABP 501 for the treatment of rheumatoid arthritis. Expert Opin. Biol. Ther..

[32] Pelechas E., Drosos A.A. (2019). Etanercept biosimilar SB-4. Expert Opin. Biol. Ther..

[33] Mueller R.B., Spaeth M., von Restorff C., Ackermann C., Schulze-Koops H., von Kempis J. (2019). Superiority of a Treat-to-Target Strategy over Conventional Treatment with Fixed csDMARD and Corticosteroids: A Multi-Center Randomized Controlled Trial in RA Patients with an Inadequate Response to Conventional Synthetic DMARDs, and New Therapy with Certolizumab Pegol. J. Clin. Med..

[34] Taylor P.C., Balsa Criado A., Mongey A.B., Avouac J., Marotte H., Mueller R.B. (2019). How to Get the Most from Methotrexate (MTX) Treatment for Your Rheumatoid Arthritis Patient?-MTX in the Treat-to-Target Strategy. J. Clin. Med..

[35] Fleischmann R., Tongbram V., van Vollenhoven R., Tang D.H., Chung J., Collier D., Urs S., Ndirangu K., Wells G., Pope J. (2017). Systematic review and network meta-analysis of the efficacy and safety of tumour necrosis factor inhibitor-methotrexate combination therapy versus triple therapy in rheumatoid arthritis. RMD Open.

[36] Peper S.M., Lew R., Mikuls T., Brophy M., Rybin D., Wu H., O’Dell J. (2017). Rheumatoid Arthritis Treatment After Methotrexate: The Durability of Triple Therapy Versus Etanercept. Arthritis Care Res..

[37] Quach L.T., Chang B.H., Brophy M.T., Soe Thwin S., Hannagan K., O’Dell J.R. (2017). Rheumatoid arthritis triple therapy compared with etanercept: Difference in infectious and gastrointestinal adverse events. Rheumatology.

[38] Kaltsonoudis E., Pelechas E., Voulgari P.V., Drosos A.A. (2019). Unmet needs in the treatment of rheumatoid arthritis. An observational study and a real-life experience from a single university center. Semin. Arthritis Rheum..

[39] Boers M., Verhoeven A.C., Markusse H.M., van de Laar M.A., Westhovens R., van Denderen J.C., van Zeben D., Dijkmans B.A., Peeters A.J., Jacobs P. (1997). Randomised comparison of combined step-down prednisolone, methotrexate and suphasalazine with Sulphasalazine alone in early rheumatoid arthritis. Lancet.

[40] Landewe R.B., Boers M., Verhoeven A.C., Westhovens R., van de Laar M.A., Markusse H.M., van Denderen J.C., Westedt M.L., Peeters A.J., Dijkmans B.A. (2002). COBRA combination therapy in patients with early rheumatoid arthritis: Long-term structural benefits of a brief intervention. Arthritis Rheumatol..

[41] Van Tuyl L.H., Boers M., Lems W.F., Landewé R.B., Han H., van der Linden S., van de Laar M., Westhovens R., Van Denderen J.C., Westedt M.L. (2010). Survival, comorbidities and joint damage 11 years after the COBRA combination therapy trial in early rheumatoid arthritis. Ann. Rheum. Dis..

[42] Poppelaars P.B., van Tuyl L.H.D., Boers M. (2019). Normal mortality of the COBRA early rheumatoid arthritis trial cohort after 23 years of follow-up. Ann. Rheum. Dis..

[43] Van Vollenhoven R.F., Geborek P., Forslind K., Albertsson K., Ernestam S., Petersson I.F., Chatzidionysiou K., Bratt J., Swefot study group (2010). Conventional combination treatment versus biological treatment in methotrexate-refractory early rheumatoid arthritis: 2 year follow-up of the randomised, non-blinded, parallel-group Swefot trial. Lancet.

[44] O’Dell J.R., Mikuls T.R., Taylor T.H., Ahluwalia V., Brophy M., Warren S.R., Lew R.A., Cannella A.C., Kunkel G., Phibbs C.S. (2014). Therapies for active rheumatoid arthritis after methotrexate failure. N. Eng. J. Med..

[45] Moreland L.W., O’Dell J.R., Paulus H.E., Curtis J.R., Bathon J.M., St Clair E.W., Bridges S.L., Zhang J., McVie T., Howard G. (2012). A randomized comparative effectiveness study of oral triple therapy versus etanercept plus methotrexate in early aggressive rheumatoid arthritis: The treatment of Early/Aggressive Rheumatoid Arthritis Trial. Arthritis Rheumatol..

[46] Bathon J.M., McMahon D.J. (2013). Making rational treatment decisions in rheumatoid arthritis when methotrexate fails. N. Engl. J. Med..

[47] European Medicines Agency. https://www.ema.europa.eu/en/search/search.

